# *Ehrlichia* Infections, North Carolina, USA, 2016

**DOI:** 10.3201/eid2411.180496

**Published:** 2018-11

**Authors:** Ross M. Boyce, Alan M. Sanfilippo, John M. Boulos, Meghan Cleinmark, John Schmitz, Steve Meshnick

**Affiliations:** University of North Carolina at Chapel Hill, Chapel Hill, North Carolina, USA

**Keywords:** Tick, Ehrlichia, Rickettsia, tickborne diseases, bacteria, vector-borne infections, North Carolina, USA, United States

## Abstract

Nearly two thirds of persons suspected of having tickborne illness in central North Carolina, USA, were not tested for *Ehrlichia*. Failure to test may have resulted in a missed diagnosis for ≈13% of these persons, who were therefore substantially less likely to receive antimicrobial treatment and to have follow-up testing performed.

Incidence of spotted fever group *Rickettsia* (SFGR), which include the causative agent of Rocky Mountain spotted fever (RMSF), is high in North Carolina, USA (*1*). Entomologic studies, however, suggest that the principal vector in this state is the lone star tick (*Amblyomma americanum*), which is a major vector for *Ehrlichia chaffeensis* and *E. ewingii* (*2*,*3*). The clinical spectrum of *Ehrlichia* infection, which causes nonspecific signs and symptoms including fever, headache, and malaise, resembles that of RMSF (*4*,*5*). Therefore, we examined clinical practice patterns associated with the evaluation and treatment of patients suspected of having tickborne illness to determine if *Ehrlichia* infection causes underrecognized tickborne illness in North Carolina.

## The Study

We performed a retrospective chart review of all patients who had undergone serologic testing for tickborne illness at University of North Carolina hospitals and associated clinics during June 8–September 8, 2016. We abstracted demographic information, test results, and treatment plans for patients with signs and symptoms consistent with acute infection (i.e., fever, headache, myalgia) or recent tick exposure. Patients were excluded if testing had been ordered for chronic symptoms, including fatigue or neurocognitive deficits. Testing for *Borrelia burgdorferi* (Lyme disease) was performed by chemiluminescent immunoassay (Diasorin, Inc., Stillwater, MN, USA). Indirect immunofluorescence antibody (IFA) testing for SFGR was performed by using *R. rickettsia*–coated slides and a polyvalent conjugate, whereas testing for *Ehrlichia* was performed by using an IgG-based IFA (both from Biocell Diagnostics Inc, Baltimore, MD, USA). If testing for *Ehrlichia* was not ordered at the time of the initial healthcare visit, this testing was performed on stored serum (retrospective testing). Given potential cross-reactivity, we also tested retrospective samples for *Anaplasma phagocytophilum*. A positive test was defined as a positive IFA result with a subsequent IgG titer of >80 for SFGR and >64 for *Ehrlichia*.

We compared characteristics of the cohort by using the Student *t*-test for continuous variables and the Pearson χ^2^ test for categorical variables. We fit univariable Poisson regression models to examine associations between doxycycline prescriptions and test results. The study was approved by the University of North Carolina institutional review board.

We screened 226 records, from which 194 patients were included in the analysis (Table). The most common reason for patient exclusion was having been tested in response to longstanding symptoms (Figure). Tick exposure was documented for 61 (61.6%) of 99 encounters, although many providers did not record exposure history. The most commonly reported signs and symptoms were fever (38.1%), headache (27.8%), and myalgia (21.7%). The median duration of symptoms for those reporting an illness was 6 days (interquartile range 3–14 days).

**Table:** Patient characteristics stratified by provider-ordered *Ehrlichia t*esting, North Carolina, 2016*

Characteristic	Tested for *Ehrlichia*, no. (%), n = 70†	Not tested for *Ehrlichia*, no. (%), n = 124†	p value
Sex			
M	39 (55.7)	64 (52.0)	0.48
F	31 (44.2)	60 (48.0)	
Setting			0.09
Outpatient clinic	42 (63.4)	57 (48.3)	
Emergency department	16 (24.2)	47 (39.8)	
Inpatient	8 (12.1)	14 (11.9)	
Provider medical specialty			**0.001**
Emergency medicine	13 (18.8)	43 (34.7)	
Family medicine	16 (23.2)	20 (16.1)	
Infectious diseases	9 (13.0)	2 (1.6)	
Internal medicine	15 (21.7)	18 (14.5)	
Other	16 (23.2)	41 (33.1)	
Reported signs and symptoms			
Fever	27 (38.6)	47 (37.9)	0.93
Headache	22 (31.4)	32 (25.8)	0.40
Myalgia	11 (15.7)	31 (25.0)	0.13
Rash	12 (17.1)	27 (21.8)	0.44
Tick exposure‡	28 (41.2)	33 (27.7)	**0.05**
Testing			
SFGR	62 (88.6)	92 (74.2)	**0.02**
Lyme disease	48 (68.6)	80 (64.5)	0.57
Positive test result			
* Ehrlichia*	9 (12.9)	25 (20.2)	0.20
SFGR	15 (24.2)	22 (23.9)	0.97
Lyme disease	0 (0.0)	1 (1.3)	0.44
Doxycycline prescribed	27 (39.7)	67 (55.8)	**0.03**
Convalescent-phase serology performed	15 (21.7)	10 (8.1)	**0.007**

*Boldface indicates significance at p<0.05. IQR, interquartile range; SFGR, spotted fever group *Rickettsia*.  †Among those tested for *Ehrlichia*, median (IQR) age was 53.5 (33–67) y, and among those not tested for *Ehrllichia*, median (IQR) age was 46 (30–61.5) y (p = 0.48). Among those tested for *Ehrlichia*, duration of symptoms was 6.5 (3–14) d, and among those not tested for *Ehrlichia*, duration was 5.5 (3–14) d (p = 0.59). Numbers and percentages vary because some data were not available. ‡Unable to determine exposure status for 24 (35.3%) of 68 patients tested for *Ehrlichia* and 64 (53.8%) of 119 patients not tested for *Ehrlichia*.

**Figure:**
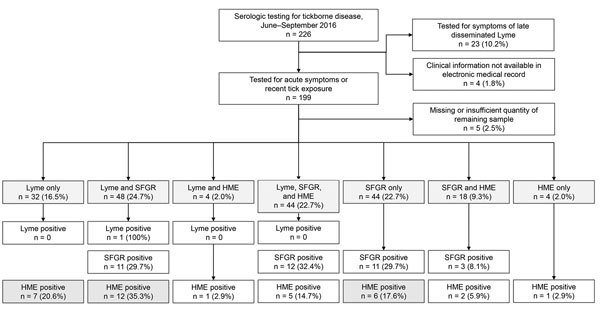
Summary of cohort selection and initial diagnostic testing results (light gray boxes) for 226 patients who had undergone serologic testing for tickborne illness, North Carolina, USA, in 2016. Results of retrospective testing for *Ehrlichia* are shown in dark gray boxes. HME, human monocytic ehrlichiosis; Lyme, Lyme disease; SFGR, spotted fever group rickettsiosis.

Most (91.8%) patients were seen in ambulatory clinics and emergency departments. Overall, most patients were tested for SFGR (154, 79.4%) and Lyme disease (128, 66.0%), but testing for *Ehrlichia* was ordered for only 70 (36.1%) patients.

A total of 154 patients were initially tested for SFGR and results for 37 (24.0%) were positive; 70 patients were initially tested for *Ehrlichia* and results for 9 (12.9%) were positive. Only 1 Lyme disease test result was positive. Of the 124 patients who did not initially have *Ehrlichia* testing performed, retrospective testing results were *Ehrlichia* positive for 25 (20.2%); none were positive for *Anaplasma*. Among those with a positive retrospective test result for whom results of a complete blood count or transaminase levels were available, 2 (12.5%) of 16 had concurrent thrombocytopenia and 1 (10.0%) of 10 had elevated transaminase levels. Convalescent-phase serologic testing results were obtained for 24 (12.5%) patients, among whom there was only 1 occurrence of a 4-fold titer increase in a patient with positive *Ehrlichia* titers. Convalescent-phase serologic testing was more frequently ordered for patients for whom acute-phase serologic results for SFGR, *Ehrlichia*, or both were positive (19/24, 79.2%).

Doxycycline was prescribed for half of all patients for whom treatment was known (94/188, 50.0%). Patients for whom acute SFGR serologic results were positive were more likely than those whose results were negative to receive doxycycline (odds ratio [OR] 7.52, 95% CI 2.73–20.8; p<0.001), but this finding was not true with regard to patients with positive versus negative results for *Ehrlichia.* Of note, doxycycline was prescribed less frequently for patients with a positive retrospective *Ehrlichia* test result (30.4%) than for those with a positive provider-ordered test (77.8%) (OR 0.13, 95% CI 0.02–0.78; p = 0.03). Similarly, convalescent-phase serologic testing was performed less often for patients with a positive retrospective test result (16.0%) than for those with a positive provider-ordered test result (55.6%) (OR 0.15, 95% CI 0.03–0.85; p = 0.03).

Our results demonstrate that *Ehrlichia* accounted for a large proportion of reactive antibodies among a cohort of patients suspected of having tickborne illness in central North Carolina. These findings provide strong, albeit circumstantial, evidence that *Ehlichia* infection is as prevalent as SFGR infection. Yet, providers ordered *Ehrlichia* testing much less frequently than SFGR or even Lyme disease testing, despite the low incidence of Lyme disease in the state. This disparity may be attributable to unfamiliarity with local vector epidemiology and to the greater attention given to RMSF and Lyme disease by the general population.

Our results show that testing strategies had a clear effect on patient care. Despite the recommendation that doxycycline be empirically given to patients suspected of having RMSF, our findings show that providers were significantly more likely to prescribe doxycycline when the acute-phase serologic test results were reactive (*6*). By extension, because providers were ordering testing for *Ehrlichia* less frequently, patients who were ultimately found to have positive retrospective serologic results were not identified during routine evaluation and thus were less likely to receive antimicrobial therapy.

Our study has several limitations, the most relevant of which is the reliance on single time point serologic testing for most patients. The absence of convalescent-phase serologic testing adversely affects our ability to discriminate acute infection from prior exposure. The presence of thrombocytopenia or elevated transaminase levels suggests that at least a portion of patients found to have reactive antibodies by retrospective testing had acute infections, but testing for these laboratory abnormalities was not performed for all patients. Thus, we may have misclassified some prior exposures as acute infections and some acute infections as noninfections.

Further complicating the picture is the issue of cross-reactivity, especially between members of SFGR, such as *Rickettsia rickettsii* and *R. parkeri*, and potentially with the newly classified *R. amblyommatis* (*7*), which is also transmitted by lone star ticks. The use of PCR for the diagnosis of *Ehrlichia* infection could have overcome issues related to *E. ewingii* cross-reactivity but was not routinely ordered and could not be performed on stored serum. We did, however, perform IFA testing for *A.* *phagocytphilum* on retrospective samples to ensure no cross-reactivity with *Ehrlichia*.

Our sampling strategy probably did not capture all suspected cases in the specified period. Patients with a compelling history and clinical signs and symptoms may have empirically received doxycycline without testing. This practice could have biased our sample such that the patients most likely to have acute infection were treated empirically, whereas testing was only performed only for those with less typical presentations.

## Conclusions

Our results demonstrate that, when considering tickborne illnesses, providers in North Carolina consider ehrlichiosis less frequently than RMSF and Lyme disease, despite the relatively high seroprevalence of antibodies reactive against *Ehrlichia* spp. in our cohort. Statewide education efforts targeting primary care offices and emergency departments are needed to improve provider awareness of and approaches to this potentially severe disease. Given the wide and growing distribution of lone star ticks, these findings are probably generalizable to much of the mid-Atlantic United States (*8*).
